# Can COVID‐19 Lead to Refractory Thrombotic Thrombocytopenic Purpura (TTP) During Pregnancy and Postpartum? A Case Report and a Review of the Literature

**DOI:** 10.1002/ccr3.70878

**Published:** 2025-09-08

**Authors:** Azadeh Shabani, Marjan Razi‐Khosroshahi, Hamide Rahmani Seraji, Nafiseh Faghih, Matin Hooshyar

**Affiliations:** ^1^ Preventive Gynecology Research Center (PGRC) Shahid Beheshti University of Medical Sciences Tehran Iran; ^2^ Resident of Obstetrics & Gynecology, Preventive Gynecology Research Center (PGRC) Shahid Beheshti University of Medical Sciences Tehran Iran; ^3^ Department of Hematology and Oncology, Taleghani Hospital Shahid Beheshti University of Medical Science Tehran Iran; ^4^ School of Medicine Shahid Beheshti University of Medical Sciences Tehran Iran

**Keywords:** COVID‐19, microangiopathic hemolytic anemia, pregnancy, thrombotic thrombocytopenic purpura

## Abstract

Thrombotic thrombocytopenic purpura (TTP) is a rare but fatal blood disorder characterized by microangiopathic hemolytic anemia, thrombocytopenia, and multi‐organ dysfunction. This condition often worsens during pregnancy and the postpartum period due to physiological changes. The present study reports a case of refractory TTP in a pregnant woman with a history of positive COVID‐19 infection who required aggressive treatment throughout her pregnancy and postpartum recovery. A 41‐year‐old pregnant woman (G3P2L2; C/S × 2) presented at 6 weeks of gestation with sudden hematuria, fever, and a positive COVID‐19 PCR test. Laboratory findings revealed the presence of schistocytes on the blood smear and a platelet count of 10,000/μL, strongly suggestive of TTP. Following hematology and gastroenterology evaluations, treatment was initiated immediately with methylprednisolone (1 mg/kg) and plasma exchange (PLEX). At 11 weeks of gestation, the patient's platelet count decreased to 12,000/μL, with evidence of hemolysis. She underwent eight sessions of PLEX and continued oral prednisolone therapy (1 mg/kg). The patient was subsequently discharged with a platelet count of 300,000/μL. Weekly outpatient PLEX and oral prednisolone were maintained throughout the pregnancy. At 36 weeks of gestation, the patient presented with labor pain and headache, leading to an emergency cesarean section. Postoperatively, she underwent 10 additional PLEX sessions and continued oral prednisolone treatment. She was discharged after 2 weeks in stable condition, without further complications. In conclusion, post‐COVID‐19 TTP could affect women and poses substantial therapeutic challenges. This case study highlights the complexities involved in managing refractory TTP during pregnancy and the postpartum period.

**Summary**

- COVID‐19 can cause refractory thrombotic thrombocytopenic purpura (TTP) during pregnancy and the postpartum period.
- Recognition of this potential association is essential for timely diagnosis and effective management.

## 1 Introduction

Thrombotic thrombocytopenic purpura (TTP) is a rare, life‐threatening condition, affecting approximately 10 individuals per million in the general population [1]. Pregnancy induces a prothrombotic state, which can complicate the diagnosis and management of TTP. While this condition typically occurs during the third trimester of pregnancy, it can, though rarely, present early in pregnancy, posing significant clinical challenges for both the mother and the fetus [2].

Since the emergence of COVID‐19, various coagulation disorders have been reported in recovering patients. These coagulopathies include deep venous thrombosis, pulmonary thromboembolism, and thrombotic microangiopathies such as TTP and disseminated intravascular coagulation. The exact mechanisms behind these disorders remain unknown; however, TTP is believed to involve an autoimmune response characterized by the presence of autoantibodies targeting ADAMTS13 [3, 4].

Diagnosis of TTP is primarily clinical and relies on laboratory findings. Common symptoms include skin rashes, fever, pale skin, confusion, and headaches. Laboratory findings typically show microangiopathic hemolytic anemia, thrombocytopenia, and multi‐organ dysfunction, with microangiopathic hemolytic anemia being the most classic finding in TTP [5]. However, the absence of obvious symptoms in many patients underscores the importance of rigorous laboratory assessment.

Thrombotic thrombocytopenic purpura mainly arises from a deficiency or inhibition of ADAMTS13—a metalloproteinase responsible for cleaving a significant portion of von Willebrand factor (vWF). The standard treatment for TTP is plasma exchange (PLEX) therapy, which aims to increase the patient's ADAMTS13 levels [6]. Notably, most cases of TTP experience recurrent episodes.

The current case report discusses a 41‐year‐old pregnant woman who was diagnosed with TTP at 6 weeks of pregnancy. She underwent multiple sessions of positron emission tomography and immunosuppressive therapy. This report highlights the critical importance of early diagnosis of TTP and appropriate intervention to address complications associated with refractory TTP during pregnancy and the postpartum period.

## 2 Case History

A 41‐year‐old pregnant woman (G3P2L2 C/S × 2) presented to Ayatollah Taleghani Hospital, Tehran, Iran, at 6 weeks of gestation, due to sudden hematuria and fever. At the time of admission, a nasopharyngeal swab confirmed COVID‐19 infection through a positive RT‐PCR test. Physical examination indicated conjunctival pallor, but there were no signs of rash, purpura, or petechiae. Neurological assessment showed no abnormalities. Her vital signs were as follows: pulse rate of 98 bpm, blood pressure of 112/76 mmHg, body temperature of 38.5°C, and respiratory rate of 19 beats per min (bpm).

### 2.1 Differential Diagnosis, Investigations, and Treatment

Initial laboratory findings demonstrated that the patient had a hemoglobin (Hb) level of 8.5 g/dL and a platelet count of 12,000/μL. Additional findings exhibited mildly elevated liver enzymes, along with proteinuria and hematuria on urinalysis. Total bilirubin was elevated at 3.1 mg/dL, creatinine measured 0.7 mg/dL, haptoglobin level was reduced to 0.151 mg/dL, and lactate dehydrogenase (LDH) was significantly elevated at 1692 U/L. Fibrinogen levels were recorded at 389 mg/dL, D‐dimer at 2588 μ/mL, and the reticulocyte production index (RPI) at 1.5. Peripheral blood smear showed normocytic anemia and thrombocytopenia, with the presence of schistocytes (Table 1). Abdominal ultrasound revealed normal results. Clinically, the patient reported flu‐like symptoms, including rhinorrhea and low‐grade fever, persisting for the past 2 weeks. While no additional diagnostic tests were performed, her clinical presentation was suggestive of a COVID‐19 infection. The presence of schistocytes on the blood smear, a critical finding in microangiopathic hemolytic anemia, strongly supported the diagnosis of TTP. ADAMTS13 antibody testing was initiated; however, treatment was immediately started due to high clinical suspicion, without awaiting the results. Following hematology and gastroenterology consultations, the patient received immediate therapy consisting of methylprednisolone (1 mg/kg) and PLEX. She underwent four sessions of PLEX therapy. After 3 days of treatment, her platelet count increased to 152,000/μL, and her Hb level stabilized at 10 g/dL. The patient was subsequently discharged, with follow‐up appointments arranged with obstetrics and hematology within 3 days.

**TABLE 1:** Laboratory findings obtained following the patient's admission at a gestational age of 6 weeks.

Laboratory tests		Normal range
Na	138 meq/L	135–145
K	3.5 meq/L	3.5–5
PT	13.6 s	10–13.8
INR	1.09	0.8–1.12
PTT	29.7 s	20–40
Uric acid	3.9 mg/dL	3.6–8
AST	69 iU/L	Up to 38
ALT	27 iU/L	Up to 42
AlkP	119 U/L	64–306
Ca	8.3 mg/dL	8.5–10.5
Ph	2.2 mg/dL	2.5–5
Mg	1.6 mg/dL	1.5–2.5
CRP	46.9 mg/L	Up to 6
Anti ds DNA	12.4 Iu/mL	Negative: Up to 30
		Equivocal: 30–35
		Positive: > 35
ANA	< 1/100	Negative: < 1/100
WBC	7900/dL	4000–11,000
Hemoglobin	9.8 g/dL	12–16
MCV	89.16 fL	80–102
PLT	12,000/μL	160–450
RPI	1.5%	0.5%–2.5%
Lactate	12 mg/dL	Arterial Blood: 8.1–15.3
		Venous Blood: 5.5–14.5
LDH	2607 U/L	230–480
Urine analysis	Protein +++, Blood ++++, WBC = 2–3, RBC = 20–45	
PBS	Schistocyte: +, Giant PLT: +	
COVID‐19 PCR	Positive	

### 2.2 Patient's Outcome and Follow‐Up

**FIGURE 1:**
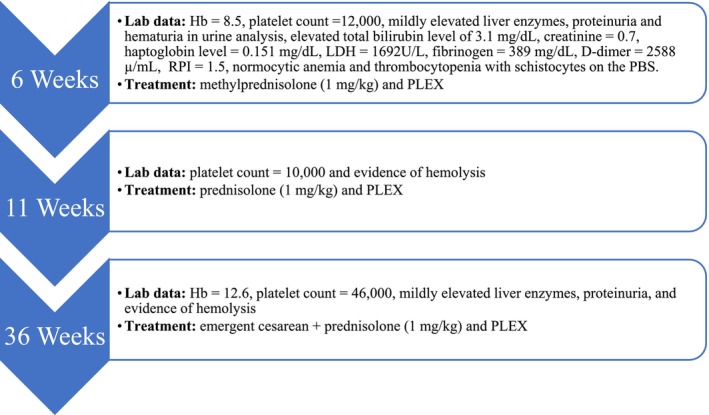
Timeline of clinical relapses with corresponding laboratory findings and treatments from 6 to 36 weeks of gestation. The figure summarizes hemoglobin and platelet levels, evidence of hemolysis, additional laboratory abnormalities, and the interventions used at each relapse episode, including corticosteroid therapy, plasma exchange (PLEX), and emergency delivery.

The patient returned 1 month later, at 11 weeks of gestation, with a decreased platelet count of 10,000/μL and signs of hemolysis. She received four sessions of PLEX therapy and was also prescribed oral prednisolone at a dose of 1 mg/kg. Following the treatment, her platelet count increased to 300,000/μL (Table 2), and she was discharged with a plan for weekly outpatient PLEX and ongoing oral prednisolone therapy (1 mg/kg). At 36 weeks of gestation, the patient was admitted due to labor pains, headaches, nausea, vomiting, and suicidal thoughts. Upon examination, her pulse was 96 bpm, blood pressure was 150/110 mmHg, body temperature was 37.5°C, and respiratory rate was 19 bpm. Initial laboratory findings showed a Hb level of 12.6 g/dL, a platelet count of 46,000/μL, mild elevation in liver enzymes, presence of proteinuria, and evidence of hemolysis (Table 3). Given the diagnosis of TTP and severe pre‐eclampsia, an emergency cesarean section was performed. The patient underwent eight additional sessions of PLEX, while continuing her prednisolone treatment at 1 mg/kg. Following surgery, a gradual normalization of her platelet count, LDH levels, and liver enzymes was observed. However, on Day 10 post‐operation, the patient developed epigastric pain accompanied by a sudden increase in liver enzyme levels. An abdominal sonography revealed distention of the common bile duct, along with biliary sludge and small stones, measuring up to 32 mm in diameter, within the posterior shadow. A cholangiopancreatography was conducted, and the findings were normal. Eventually, her liver enzyme levels began to normalize, and she was discharged in stable condition without any complications (Figure 1).

**TABLE 2:** Laboratory findings obtained following the patient's admission at a gestational age of 11 weeks.

Laboratory tests		Normal range
PT	10.4 s	10–13.8
INR	0.95	0.8–1.12
PTT	25.6 s	20–40
WBC	8800/dL	4000–11,000
Hemoglobin	5.6 g/dL	12–16
MCV	104.9 fL	80–102
PLT	100,000/μL	160–450
RPI	2.38%	0.5%–2.5%
LDH	580 U/L	230–480
ADAMTS13 activity	19%	40%–130%
PBS	Schistocyte: 2+	
	Polychromasia: 3+	
	Anisocytosis: 2+	

**TABLE 3:** Laboratory findings obtained following the patient's admission at a gestational age of 36 weeks.

Lab tests		Normal range
AST	39 iU/L	Up to 38
ALT	20 iU/L	Up to 42
AlkP	191 U/L	64–306
WBC	11,200/dL	4000–11,000
Hemoglobin	13.6 g/dL	12–16
MCV	87.56 fL	80–103
PLT	76,000/μL	230–480
RPI	5.02%	0.5%–2.5%
ADAMTS Ab	3.9 IU/mL	Negative: < 12
		Borderline: 12–15
		Positive: > 15

### 2.3 Fetal Outcome

The newborn was delivered with APGAR scores of 6 at 1 min and 8 at 5 min, and had a birth weight of 1420 g. Due to the maternal history and low APGAR scores, the neonate was admitted to the Neonatal Intensive Care Unit (NICU) for observation and further evaluation. During the 72‐h stay in the NICU, the neonate remained clinically stable. Initial laboratory tests revealed a platelet count of 112,000/μL and a Hb level of 16.9 g/dL. There were no episodes of bleeding or organ dysfunction. The infant was discharged in good health on the third day of life.

## 3 Discussion

The present study reports a case of refractory TTP diagnosed during the first trimester of pregnancy, focusing on the therapeutic approach and outcomes for the mother and fetus.

TTP is a rare but potentially life‐threatening disorder that may arise during pregnancy, posing substantial risks to maternal and fetal health. Unlike preeclampsia and HELLP syndrome—conditions specifically associated with pregnancy—TTP is often neglected, which can result in delayed diagnosis and intervention [7]. Notably, TTP may coexist with these conditions, as demonstrated by our patient's near‐term presentation with severe preeclampsia. The differentiation between TTP and other pregnancy‐associated microangiopathies is clinically challenging due to overlapping features [8]. TTP is characterized by severely reduced or absent ADAMST13 activity, leading to widespread microvascular thrombosis and organ dysfunction. Its clinical manifestations can vary from non‐specific symptoms such as fatigue and dyspnea to petechiae, renal impairment, and neurological deficits [9].

Diagnosis of TTP is based on clinical findings, particularly thrombocytopenia and microangiopathic hemolytic anemia. Definitive confirmation requires demonstrating reduced ADAMTS13 activity—mainly < 10%—and the presence of an ADAMTS13 inhibitor in an appropriate clinical setting. During the diagnostic process, excluding other autoimmune disorders, such as lupus, is essential. Routine ADAMTS13 testing is not an immediate diagnostic tool, as processing time can delay results and may not provide rapid clinical guidance [10]. Viral infections are recognized as prominent triggers for TTP, acting through mechanisms such as direct endothelial injury and the induction of autoantibodies against ADAMTS13 [11] The hypercoagulable state observed in COVID‐19 has also been linked to cytopathic endothelial damage, possibly mediated via entry through the ACE‐2 receptors [12].

In the case of our study, PLEX and corticosteroids were initiated after clinical suspicion of TTP, given the elevated risk of maternal and fetal mortality. Although there was an initial rise in platelet counts following PLEX treatment, their levels declined within a few days, according to the criteria for refractory TTP—defined as an absence of platelet response after 4 to 7 days of PLEX or a clinical deterioration in a patient receiving standard therapy [13]. Given potential teratogenic effects, monoclonal antibody therapy was withheld, and PLEX was continued prophylactically [14].

## 4 Literature Review

Post‐COVID‐19 TTP mostly affects women and tends to be refractory, with patients typically requiring an average of 12 sessions of PLEX per case [15]. Emerging data suggest that COVID‐19, similar to other viral infections, can trigger TTP through direct or immune‐mediated mechanisms [16]. Infection with SARS‐CoV‐2 is known to cause significant endothelial injury, giving rise to excessive release of vWF multimers. Furthermore, the inflammatory environment associated with COVID‐19 could promote the production of autoantibodies targeting ADAMTS13, the metalloprotease responsible for cleaving ultra‐large vWF multimers [17]. This dual mechanism—marked by elevated vWF levels and reduced ADAMTS13 activity—disrupts hemostatic balance and can result in microvascular thrombosis, a central feature of TTP. In a cross‐sectional study conducted by Mancini et al. [18] involving 50 patients, median vWF levels were significantly elevated in COVID‐19 patients, which correlated with the intensity of care required. In contrast, the ratio of hightolow molecular‐weight vWF multimers and median ADAMTS13 activity decreased as clinical severity increased. A systematic review by Chaudhary et al. [15] confirmed the association between decreased ADAMTS13 activity and COVID‐19 severity. Interestingly, Jole et al. [19] examined the conformational states of vWF and ADAMTS13 in critically ill COVID‐19 patients and found an elevated vWF/ADAMTS13 ratio at admission as a predictor of mortality. Altogether, these studies emphasize the importance of monitoring coagulation parameters in COVID‐19 patients and suggest that SARS‐CoV‐2 infection can trigger TTP in susceptible individuals.

Current treatment for TTP involves multiple sessions of PLEX. While response rates to PLEX have been reported to approach 100% [20], relapse remains a significant concern, affecting about 10% to 40% of patients. Those requiring more than 4–7 sessions are typically classified as refractory. Despite limited data on the safety of PLEX during pregnancy, existing evidence indicates that risks are similar to those in the non‐pregnant population. Common side effects of PLEX include chills (1.1%–8.8%), hives (0.7%–12%), and transfusion‐related allergic reactions. Additionally, patients may experience paresthesia due to hypocalcemia (1.5%–9%), headaches (0.3%–5%), and hypotension (0.4%–4.2%) [21].

Treatment protocols for TTP recommend PLEX as the first‐line treatment [8]. However, recent studies have highlighted the use of immunosuppressive agents and monoclonal antibodies in managing refractory cases. Scully et al. [22] reported that rituximab can be used safely during pregnancy. Likewise, existing research supports the use of caplacizumab, a nanobody targeting the A1 domain of vWF, which can inhibit its interaction with platelets and thereby mitigate microvascular thrombosis. When used in combination with PLEX and immunosuppressive therapy, caplacizumab has been shown to accelerate platelet count normalization and reduce the risk of TTP‐related complications and relapse [23]. However, in our case, caplacizumab was not administered due to the lack of sufficient safety data for its use during pregnancy. Instead, early onset of TTP during the first trimester was managed successfully with continuous PLEX, which resulted in favorable outcomes for both mother and the fetus [24].

## 5 Conclusion

This case study underscores the vital importance of early diagnosis and timely intervention in managing TTP during pregnancy, particularly in the rare situation of refractory TTP occurring in the first trimester. Given the heightened risk of life‐threatening complications for both the mother and fetus, clinicians must maintain a high index of suspicion when encountering signs of microangiopathic hemolytic anemia and thrombocytopenia in pregnant patients. PLEX remains the cornerstone of treatment for TTP, and this case demonstrates its efficacy in achieving a platelet response—even in refractory cases. While monoclonal antibody therapies, such as rituximab and caplacizumab, are emerging as viable options for managing refractory TTP, their use in early pregnancy warrants careful consideration due to potential teratogenic risks. Taken together, continuous PLEX proved to be an effective treatment strategy, yielding favorable maternal and fetal outcomes. This case highlights the need for individualized treatment plans, careful monitoring, and multidisciplinary collaboration to improve prognosis in pregnant patients affected by TTP.

## Author Contributions

**Azadeh Shabani:** conceptualization, methodology. **Marjan Razi‐Khosroshahi:** conceptualization, methodology, project administration, supervision, writing – review and editing. **Hamide Rahmani Seraji:** writing – original draft, writing – review and editing. **Nafiseh Faghih:** writing – original draft. **Matin Hooshyar:** investigation.

## Consent

The patient gave written informed consent to publish this report in accordance with the journal's patient consent policy.

## Conflicts of Interest

The authors declare no conflicts of interest.

## Data Availability

Data sharing not applicable to this article as no datasets were generated or analyzed during the current study.
